# Supplementary material for: Are carbon emissions trading and green financial instruments synergistic? -Comprehensive quantitative research based on content analysis
Source: PLoS One. 2024 Mar 7;19(3):e0298601. doi: 10.1371/journal.pone.0298601 (PMC10919601; doi:10.1371/journal.pone.0298601)
Supplement: S1 Data — (ZIP) [file pone.0298601.s001.zip › Data/地级市Z_F工作报告.zip!/║╙─╧/░▓╤⌠╩╨/└┤╫╘╣1⁄2╓┌║┼í╛┬φ┐╦╩2╛▌═°í┐_2011.docx]

各位代表：

    现在，我代表市人民政府向大会报告政府工作，请予审议，并请各位政协委员提出意见。

    一、2010年及“十一五”工作回顾

    2010年，面对复杂多变的经济环境和严峻挑战，在省委、省政府和市委的正确领导下，全市上下深入贯彻落实科学发展观，坚持“四个重在”实践要领，科学把握不同阶段形势变化特点，以重点项目、产业集聚区、招商引资和城乡建设四项重点工作带动全局，实施了“三大工程”和“十大行动计划”，开展了“打好攻坚战、全力保目标”活动，努力在发展中调整，在发展中转变，在发展中增效，圆满完成了市十二届人大二次会议确定的各项目标任务，经济社会发展继续保持了好的趋势、好的态势和好的气势。

    ——综合实力大幅提升。初步统计，全市生产总值1311亿元，增长13.1%，高于全国、全省平均增速；人均生产总值25000元，增长12%。地方财政一般预算收入65.1亿元、支出140.7亿元，分别增长17.9%和15.5%。

    ——工业经济提质增量。全市规模以上工业增加值648亿元，增长19.7%，占全部工业增加值的比重达88.5%；实现利润183亿元，增长34%。11家企业入围河南省工业百强企业。

    ——粮食生产再创新高。全年粮食总产334万吨，连续8年丰产丰收。滑县连续8年获得全国粮食生产先进县标兵称号。

    ——三大需求快速增长。全社会固定资产投资895亿元，增长20.8%；城镇固定资产投资760亿元，增长22.1%。社会消费品零售总额345亿元，增长18.9%。进出口总额15.7亿美元，增长66.9%。

    ——人民生活持续改善。城镇居民人均可支配收入16394元，增长10.7%；农民人均纯收入6359元，增长13.7%。城乡收入均高于全省平均水平。

    一年来，我们主要抓了以下工作：

    （一）全力以赴保持经济持续较快发展。紧紧抓住市场开拓、投资拉动、城乡建设等关键环节，因势利导，分类施策，保持了经济平稳较快增长。着力扩大投资。继续实施项目带动战略，建立重点项目联审联批联办制度，扎实推进“重点项目双百工程行动计划”，119个省、市重点项目累计完成投资169.8亿元。积极促进消费。培育消费热点，优化消费环境，大力实施万村千乡市场工程和家电下乡工程。新建、改建农家店3000多家，升级改造城区农贸市场15个。销售家电下乡产品54.1万台（部），兑付补贴资金1.5亿元。商品房销售面积339.9万平方米，增长39.3%。强化要素保障。加大银企对接和融资力度，全年新增贷款和直接融资突破170亿元。加强煤、电、油、气、运调节和土地管理，有效保障生产要素供给。深入开展服务企业活动，发展环境进一步优化。

    （二）集中精力加快发展方式转变。正确处理保增长与调结构、促转型的关系，一手抓传统产业升级改造，一手抓新兴产业谋划培育，经济发展的可持续性进一步增强。加快产业集聚区建设。大力实施“产业集聚区建设工程”，切实抓好公共服务平台、规划展馆和基础设施建设，9个省级产业集聚区完成投资310亿元，集聚规模以上工业企业234家，实现工业总产值740亿元。内黄县产业集聚区荣获“河南省承接产业转移示范区试点”称号，汤阴县产业集聚区入选“2010中原十佳活力产业集聚区”。推动产业结构优化升级。强力推进“工业振兴工程”，改造提升钢铁、装备制造等传统产业，培育壮大新能源、电子信息等战略性新兴产业。联合国工业发展组织太阳能光伏研发基地落户我市。提高自主创新能力。积极实施“自主创新行动计划”，科技支撑经济社会发展能力进一步增强。建立健全政府质量奖励制度，一批重大科技专项取得突破。国家级高新技术企业达到11家，高新区成功晋升国家级高新区。加强节能减排。大力发展循环经济，成功举办安西循环经济试验区高层论坛。积极实施重点流域、重点行业环境综合整治，环境质量持续改善。单位生产总值能耗下降4.4%，化学需氧量和二氧化硫排放量分别控制在4.5万吨和9.8万吨以下。

    （三）持之以恒夯实农业基础。认真落实支农、惠农政策，大力实施“兴农行动计划”，农业基础地位更加巩固。加快发展现代农业。6个现代农业示范引领区全面启动。新建高效农业示范园区48个，新增亩纯效益3500元以上高效农业16万亩。新建、扩建规模龙头企业25家。新增畜禽养殖密集区66个，新建大中型规模养殖加工企业600家。全面加强农业基础设施建设。南水北调安阳段进展顺利，14座病险水库除险加固工程全部竣工，5大灌区技改工程全面完成，连续17年夺得全省农田水利基本建设“红旗渠精神杯”。扎实推进新农村建设。18个省级新农村示范村全部启动，113个新农村社区开工建设。新建大中型沼气池75座，新增沼气用户3.1万户。解决了37万人的安全饮水问题。转移培训农村劳动力1.6万人。新修硬化农村公路501公里。完成97个贫困村整村推进任务，实现4.4万农村人口脱贫。加快林业生态市建设，完成营造林28万亩、“两岭”示范性造林开发3万亩，成功创建省绿化模范城市。

    （四）坚持不懈统筹城乡发展。坚持中心城市带动，促进城乡协调，基础支撑能力不断提高。深入推进“城建三年计划”。安阳新区、安东新城加快建设，林长高速、“六桥一互通”、西南外环、中华路南北段、文峰大道东段、文明大道东段等快速推进。中轴线水系景观加快建设，洹河清淤顺利完成，洪河治理一期工程竣工通水，茶店河综合治理全线开工。数字化城管一期工程投入运行，成功创建为国家园林城市，创建国家卫生城市通过国家暗访。大力实施“现代交通体系行动计划”。京港澳高速安阳段改扩建工程完成，石武高铁安阳段、晋豫鲁铁路安阳段相继开工。豫北支线机场列入国家、省发改委计划，安阳陆路港综合服务中心挂牌成立，邯郸机场安阳航站楼正式启用。不断加快城乡一体化进程。编制完成城乡一体化空间发展战略规划，深入开展“优胜杯”竞赛活动，加快县城、小城镇和村庄建设，城乡综合承载能力进一步提高。14个乡镇入围河南省百强乡镇，水冶镇连续6年保持全省乡镇综合实力第一名。

    （五）扎实有效抓好以文化旅游为龙头的第三产业。积极推进“文化旅游行动计划”，第三产业取得新突破。文化产业快速发展。全市经营性文化单位转企改制全部完成。殷墟列入全国第一批国家考古遗址公园，曹操高陵入选省第五批文物保护单位。三杨庄汉代遗址展厅积极推进。市图书馆顺利晋升国家一级图书馆。成功举办2010中国·安阳殷商文化旅游节、“安阳杯”第四届黄河戏剧节、首届两岸汉字艺术节等大型节庆活动。旅游业持续繁荣。殷墟晋升5A级景区，红旗渠、马氏庄园列入全国红色旅游经典景区名录。内黄森林公园、龙泉生态公园、洹水湾温泉旅游区等项目进展顺利。成功举办2010年滑翔伞世界杯赛暨第一届中国安阳林滤山国际滑翔伞公开赛。荣获“国际知名旅游目的地”称号。全年接待国内外游客1885.9万人次，旅游总收入127.3亿元，分别增长31.4%和162.4%。现代服务业蓬勃发展。华强建国酒店投入运营，安阳迎宾馆加快建设。沃尔玛商场盛装开业，义乌国际商贸城、彰德府商城、高新区铁路专用线等商贸物流项目顺利推进。招商银行、中信银行落户安阳，广发证券安阳营业部挂牌营业。

    （六）坚定不移深化改革扩大开放。把改革开放作为持续发展的根本途径，以改革破解难题，以开放带动全局，不断增强经济社会发展的内生动力。重点领域改革深入推进。继续深化国企改革，基本完成40户企业改革任务。煤矿兼并重组取得重大进展，14家煤矿与省骨干煤炭企业签订正式协议。林州重机、顺成煤焦成功上市。集体林权制度、警务体制、医药卫生体制、城管体制等改革也都取得了新成绩。招商引资成效显著。把招商引资作为“一举应多变”的关键举措，围绕支柱产业和产业集聚区，积极开展“五百招商行动”、“会战长三角、集中大招商”等一系列活动。与华强、沙钢、宏威等170余家大型企业集团建立了长期友好合作关系，与中建材、中石化、华融集团等央企合作进一步深化，成功引进BD-R蓝光生产基地等亿元以上项目144个。全年引进域外资金345.6亿元，增长25%；实际利用外资1.5亿美元，增长26.6%。

    （七）千方百计保障和改善民生。在财力有限的情况下，积极筹措资金40多亿元用于“十项民生工程”，85个项目如期完成。就业工作得到加强。完善全民创业服务体系，积极创建省级创业型城市。新增城镇就业9万人，再就业3.4万人，其中困难群体再就业1.3万人。农村劳动力转移就业167万人。社会保障体系进一步完善。新农保试点工作进展顺利，工伤保险实现市级统筹，城镇职工基本医疗保险最高支付限额大幅上调，企业离退休人员基本养老金人均月增149.7元，城乡低保、优抚对象和农村“五保”补助标准进一步提高。新建经济适用住房26.4万平方米、廉租住房26万平方米，完成棚户区改造5.4万平方米，在全省率先建立了保障性住房服务大厅。加强价格管理和调控，加大低收入群体补贴力度，有效保障了群众的基本生活。社会事业全面进步。义务教育均衡发展，职教攻坚计划顺利推进，高新区在全省率先实行12年义务教育。基层医疗卫生服务体系逐步完善。公共文化服务体系建设扎实推进，文化惠民工程取得新进展。文化宫、规划馆、会展体育中心、房产交易中心建设加快实施。广泛开展全民健身活动，竞技体育水平不断提高。继续做好“放心早餐工程”，成功举办全国早餐企业年会。认真开展第六次全国人口普查。积极推进人防建设融合式发展，荣获“全国人民防空先进城市”称号。成功创建“全国人口和计划生育综合改革示范市”。人事人才工作得到加强。深入实施“安全发展行动计划”，安全生产事故起数和死亡率分别下降84.1%和73.5%。食品药品安全保障水平继续提高。推进平安安阳建设，落实信访稳定风险评估机制，社会治安综合防控体系不断完善，预防和应对突发事件能力逐步增强。

    精神文明和民主法治不断加强。坚持“三具两基一抓手”，深入推进“两转两提”，扎实开展创先争优活动，大力弘扬“三平”精神，团结拼搏、干事创业的氛围更加浓厚。自觉接受人大法律监督、工作监督和政协民主监督，认真办理人大代表议案、建议和政协委员提案。深入推进反腐倡廉建设。积极实施市民素质提升工程，公民道德水平和城乡文明程度不断提高。大力开展国防教育，扎实做好“双拥”工作。不断加强老区建设。依法保护妇女儿童合法权益。老龄和关心下一代工作健康发展。慈善事业走在全省前列。统计、审计、史志、档案、气象、地震、发展研究、外事侨务、民族宗教、红十字等工作进一步加强。圆满完成对口支援四川江油灾后重建工作。

    各位代表！

    2010年各项工作目标的实现，标志着我市“十一五”主要任务胜利完成。

    ——这五年是我市经济发展全面提速、综合实力显著增强的五年。生产总值增幅连年保持在两位数以上，经济总量由560.6亿元增加到1311亿元，人均生产总值由1.04万元增加到2.5万元，均实现了翻一番；全部工业增加值累计达到2719亿元，是“十五”时期的3倍；地方财政一般预算收入由“十五”末的29亿元增加到65.1亿元；社会消费品零售总额累计完成1250亿元，较“十五”时期增加677亿元。

    ——这五年是我市产业结构调整优化、发展方式加快转变的五年。初步统计，2010年，全市三次产业结构比为12.1∶61.7∶26.2，一产占比大幅下降，二产占比继续增加，三产占比不断提高。高新技术产业产值累计达到591亿元，年均增速保持在20%以上；第三产业增加值由“十五”末的153亿元提高到343亿元，增长96.3%。非公有制经济增加值占生产总值的比重达到56%。

    ——这五年是我市粮食产量连创新高、农业基础不断巩固的五年。改造中低产田31.2万亩，新增有效灌溉面积49.2万亩，粮食播种面积稳定在800万亩以上，粮食产量由50亿斤增加到66.8亿斤，年均净增3亿多斤，为我市经济社会发展和国家粮食安全作出了积极贡献。

    ——这五年是我市城乡面貌显著改观、对外形象明显提升的五年。城市建成区面积由83平方公里增加到102平方公里，城镇化率达到40.4%，较“十五”末提高7.9个百分点，新增城镇人口47万人，是历史上城镇化发展最快的时期。解决了110万人的饮水安全问题，新增沼气用户21万户，新农村和新型农村社区建设迈出坚实步伐。

    ——这五年是我市基础设施日益完善、支撑能力明显增强的五年。全社会固定资产投资累计达到2932亿元，年均增长31%。全市公路通车里程达到11651公里，其中，高速公路227公里，大广高速滑县段、安林高速、安南高速等建成通车，形成了铁路、高速“双十字”交通构架，区域性交通枢纽地位进一步凸显。

    ——这五年是我市社会事业全面发展、人民生活极大改善的五年。各级财政用于民生方面的支出累计达到211亿元，是“十五”时期的3.5倍。新增城镇就业累计57.3万人；农村劳动力转移就业净增60.1万人。全市普通高校在校生4.8万人，比“十五”末增加了近2万人。千人病床数3.6张，新农合参合率98.8%。城镇居民人均可支配收入由8822元提高到16394元，增加7572元；农民人均纯收入由3220元提高到6359元，增加3139元。

    “十一五”时期取得的辉煌成就，书写了我市全面建设小康社会崭新的一页，标志着安阳进入了建设豫北区域性中心强市的新阶段，也为下一步全市经济社会发展再上新台阶奠定了坚实基础。这些成绩的取得，是市委正确领导的结果，是市人大、市政协大力支持和监督的结果，是全市广大干部群众克难攻坚、共同奋斗的结果。在此，我代表市人民政府，向工作在全市各条战线上的工人、农民、知识分子、干部、解放军指战员、武警官兵、政法干警以及社会各界人士，致以崇高的敬意！向所有关心、支持我市经济社会发展的港澳台同胞、海外侨胞和国际友人，表示衷心的感谢！

    各位代表！

    五年的成绩来之不易，积累的经验十分珍贵。特别是应对国际金融危机的实践，使我们对科学发展观的认识不断深化，应对复杂困难局面的能力进一步提高。回顾“十一五”时期的各项工作，我们有以下几点深刻体会。一是必须始终坚持发展第一要务。以科学发展观统领经济社会发展全局，抢抓扩大内需、建设中原经济区以及新一轮产业转移等战略机遇，深入推进经济结构调整和产业转型升级，在发展中促转变，在转变中谋发展。二是必须始终坚持当前利益与长远发展相结合。既要立足当前扩内需、保增长，努力保持一定的发展速度，也要着眼长远打基础、增后劲，坚持长短结合，科学运作，在实现当前发展的同时为长远发展奠定基础。三是必须始终坚持深化改革扩大开放。以深化改革激发发展活力，以扩大开放拓展发展空间，以优化环境聚集发展资源，不断为经济社会发展注入强大动力。四是必须始终坚持协调联动打总体战。坚持凝聚全市力量，汇集各方智慧，群策群力，形成团结协作、合力攻坚的浓厚氛围，谋大事、办实事、解难事。五是必须始终坚持保障和改善民生。切实把保障和改善民生作为发展的出发点和落脚点，将经济发展与民生改善相结合，确保广大人民群众共享改革发展成果。

    各位代表！

    在肯定成绩的同时，我们也清醒地认识到，经济社会发展中还存在不少矛盾和问题。一是产业结构不合理，钢铁、有色、电力等传统产业仍占相当一部分比重，三产发展相对缓慢，转变经济发展方式和国企改革、产业提升、结构调整的任务比较艰巨。二是随着国家宏观调控力度加大和资源环境约束加剧，节能减排形势仍不容乐观，保持经济长期又好又快发展难度增加。三是区域经济发展不协调，城区经济滞后于县域经济。四是城乡建设项目征地、拆迁、融资压力增大，有的项目推进较慢。带动能力强的大项目、好项目不够多，不够强。五是公共服务水平与人民群众日益增长的物质文化需求仍有较大差距，面临既要加快发展又要提升公共服务和生活水平的双重压力。六是一些干部的工作方法还不适应形势需要，工作作风不够扎实，统筹运作、执行落实能力不够强。对此，我们一定高度重视，在今后的工作中认真加以解决。

    二、“十二五”发展的总体思路和主要目标

    “十二五”时期是我市全面建设小康社会的关键时期，是深化改革开放、加快转变发展方式的攻坚时期，也是实现五年新跨越的战略机遇期。纵观安阳发展，改革开放不断深入推进，发展活力和动力日趋强劲；连续多年的较快增长，经济总量和综合实力大幅提升，为加快发展、跨越发展提供了重要支撑。围绕中原经济区建设，我们明确了要把安阳建设成为新型城镇化、工业化和农业现代化协调发展示范区、中原经济区综合实力领先城市和豫北区域性中心强市的战略定位；确定了着力打造新型工业基地、现代农业示范市、豫北区域性金融中心、全国文化旅游名城、科教创新人才培养基地、区域性现代交通枢纽、晋冀豫结合部商贸物流中心、中国航空运动之都及低空经济发展试验区等八个战略重点。这些都为我市经济社会发展指明了方向，但同时也要看到，世界经济复杂多变，区域竞争日趋激烈，转型发展任重道远。全市上下必须切实增强忧患意识和责任意识，抢抓机遇、积极有为，凝心聚力、励精图治，奋力实现经济社会跨越式发展。

    “十二五”时期，政府工作的总体思路是：高举中国特色社会主义伟大旗帜，以邓小平理论和“三个代表”重要思想为指导，深入贯彻落实科学发展观，以科学发展为主题，以加快转变经济发展方式为主线，以富民强市为中心任务，牢牢抓住建设中原经济区的战略机遇，紧紧扭住工业、抓好农业、加快发展第三产业，大力实施项目带动战略，强化产业支撑、城市建设支撑、交通运输支撑、软实力支撑，把我市打造成富强、宜居、枢纽、创新、和谐的豫北区域性中心强市。

    综合考虑未来发展趋势和条件，今后五年我市经济社会发展的主要目标是：

    ——经济发展水平明显提高。2015年，生产总值达到2400亿元，主要经济指标年均增速高于全省、人均水平高于全省，民生指标优于全省、效益指标优于全省；科技创新能力增强，经济结构明显优化，经济发展方式明显转变，经济发展的质量和效益明显提升。

    ——“三化”协调发展取得明显成效。新型工业化步伐明显加快，农业基础地位更加牢固，城乡一体化发展格局加快形成，城镇化处于全省先进水平。

    ——生态环境明显改善。防灾减灾体系更加完善，资源利用效率显著提高，主要污染物减排成效明显，资源节约型和环境友好型社会建设取得实质性进展。

    ——改革开放明显推进。重点领域和关键环节改革取得突破，开放型经济达到新水平，承接产业转移取得显著成效。

    ——社会建设明显加强。民生事业不断发展，就业岗位持续增加，城乡居民收入水平普遍较快增长，生活质量明显提高，公共服务体系和社会保障体系逐步完善；公民思想道德素质、科学文化素质和健康素质不断提高；文化事业、文化产业和文化旅游发展明显加快；民主法制和精神文明建设进一步加强，社会大局保持稳定。

    《安阳市国民经济和社会发展第十二个五年规划纲要（草案）》及其说明已印发各位代表，请予审议。

    三、2011年工作安排

    2011年是实施“十二五”规划的开局之年，做好今年的工作意义重大。必须坚持省委提出的“四个持续”、“四个着力”、“四个突出”，大力实施市委、市政府提出的“八大行动计划”，确保开好局、起好步。

    今年经济社会发展主要预期目标是：生产总值增长12%以上；地方财政一般预算收入增速高于经济增长速度；全社会固定资产投资增长20%，其中，城镇固定资产投资增长22%以上；新增城镇就业6.5万人以上，城镇登记失业率控制在4.5%以内；城乡居民收入增长高于全省平均水平；人口自然增长率控制在6.3‰以内；居民消费价格涨幅控制在4%左右；社会消费品零售总额、出口总额、实际利用外资和节能减排完成省定目标。

    在实现上述目标的同时，市委、市政府还要全力实施“十项民生工程”，请一并审议。

    今年着重抓好以下八个方面工作：

    （一）着力扩大需求，开创科学发展新局面。扩大需求规模，优化需求结构，为推动经济平稳较快发展创造条件。加强重点项目建设。以新兴产业、交通能源、社会事业等领域为重点，集中力量建设一批基础设施和转型升级项目。突出抓好安钢冷轧、新能光伏、凤凰光伏、一汽专用汽车、中原瓷都、市职业教育中心等127个投资规模大、带动能力强的重点项目。完善分级负责、三级联动、部门联审联批联办项目推进机制。积极争取中央、省投资项目。着力扩大消费需求。落实调整收入分配关系的各项政策，努力提高城乡低收入群体收入，扩大中等收入群体，增强居民消费能力。大力发展物流中心、大型商场，继续实施万村千乡市场工程，进一步做好家电下乡和以旧换新工作。基本完成城区农贸市场改造任务。积极发展文化旅游消费、休闲健身消费，满足群众多样化消费需求。大力整顿和规范市场秩序，营造良好的消费环境。健全企业服务长效机制。大力实施“支持服务央属、省属企业行动计划”，建立健全政策导向、市场开拓、融资服务、困难企业帮扶等长效机制，重点抓好高成长性企业和经济效益规模企业。加强经济运行调节和监测。做好煤电油气运协调工作，保障要素供应。深入推动产销对接，积极引导上下游企业建立战略联盟。落实价格监管措施，保持市场物价基本稳定。

    （二）调整优化产业结构，构建科学发展新格局。大力实施“传统产业升级行动计划”和“新兴产业倍增行动计划”，推动产业结构转型升级，构建现代产业体系。积极推进产业集聚区建设。全面落实支持产业集聚区发展的政策措施，按照“四集一转”要求，继续完善、创新推进机制和管理办法，加强基础设施和公共服务平台建设。围绕产业集聚区功能定位，加快形成特色主导产业，组织实施一批招商引资和承接产业转移项目。调整优化产业结构。积极实施“工业振兴工程百亿元园区、百亿元产业、百亿元企业行动计划”，力争年内100亿元以上企业达到4家，50亿元至100亿元企业达到6家。改造提升冶金建材、煤化工、装备制造、食品、纺织等五大传统产业，突出发展新能源、生物医药、高端装备制造等支柱产业，加快培育新能源汽车、新材料和节能环保等战略性先导产业。重点抓好凯瑞数码150条蓝光生产线、安化20万吨乙二醇、超越集团年产180万吨煤矿等投资10亿元以上的重大项目。积极推进安彩光伏玻璃、红日锂电池正负极材料、嘉能能源二甲醚、东泰制药、精密机床等项目，优化提升产业层次。着力提升自主创新能力。新建15个至20个市级以上工程技术中心，其中省级以上2个至3个。新建5个市级以上重点实验室，支持中棉所国家重点实验室建设和安阳师院、安阳工学院等单位申报建设国家、省重点实验室。坚持质量兴市，引导激励企业积极争创省长质量奖、市长质量奖、名牌优质产品和著名商标。持续推进节能减排。完善节能减排目标责任考核评价体系，健全激励约束机制，加快淘汰落后产能、技术改造升级和产品结构调整。大力发展循环经济，加快安西循环经济试验区建设。加强环境保护，积极推进重点区域、重点流域和行业环境综合整治。

    （三）大力发展现代农业，拉长科学发展新链条。积极实施“现代农业行动计划”，确保粮食安全，促进农民增收。抓好六大现代农业示范引领区建设。重点抓好特色产业培育、重点项目实施及示范工程建设，加快提升安阳县农林水畜综合开发、内黄县“瓜果菜”高效农业、滑县粮食产业、汤阴县农业产业化、林州市大中型沼气循环农业、龙安区花木观光休闲等六大引领区建设水平，加大100万亩农业现代化试验示范区建设力度。提高粮食综合生产能力。抓好粮食生产核心区、国家优质粮产业工程建设，积极开展粮食高产创建工作，全面提升粮食综合生产能力，打造全国粮食优质高产示范基地。加快农业结构调整步伐。大力发展特色高效农业和休闲农业，新建“百、千、万”高效农业示范园区30个，新增亩纯效益3500元以上的高效农业16万亩。扩大土地流转规模，完善县、乡两级土地流转服务中心和服务大厅建设。继续实施“552”现代畜牧业发展规划，新增畜禽养殖密集区50个。大力发展农业产业化龙头企业，重点打造10个至20个农业产业化强乡镇。抓好新一轮市长负责制的“菜篮子”工程。加快水利改革发展。贯彻落实好中央一号文件精神，加大资金投入，加强引黄工程建设、大型灌区续建配套、中小河流治理和小型水库除险加固。深化水利工程管理体制改革，确保长久发挥效益。继续推进农村饮水安全工程建设，解决25万人饮水安全问题。扎实推进新农村建设。力争18个省级新农村示范村完成70%以上，14个市级“六位一体”整体推进示范区全面完成。加快农村沼气建设，积极创建全国沼气示范市。新建改建农村公路150公里。提高生态建设质量，加快城乡绿化一体化步伐，争创全国绿化模范城市。

    （四）统筹城乡协调发展，探索科学发展新路子。大力实施“加快中心城区建设和城区经济发展行动计划”，积极构建现代城镇体系。全力推进“城建三年计划2011年行动计划”。完成中华路北段、文源街等项目建设，开工建设永明路南段、文峰大道西段等12条道路，抓好人民大道、文明大道等5条城市主干道亮化工程。推进八中路口、南大街、东方红片区等5个区域的改造。开工建设具有文体活动、科技复合功能的场馆。实施洹河水系景观、东区中轴线水系建设工程，完成洪河、茶店河年度治理任务，加快南水北调蓄水池及管线配套工程建设。着力构建综合交通体系。加快石武高铁安阳段、晋豫鲁铁路安阳段、林长高速、鹤辉高速等道路建设，启动西北环城高速公路前期工作。推进107国道、301省道等干线公路升级改造。加快文昌大道、长江大道、创业大道等建设。完善陆路港、航空服务港功能，加快航校搬迁和豫北支线机场规划建设。加强城市基础设施建设与管理。积极推进城市园林绿化、公共交通、供水、供热、燃气、污水、垃圾处理等基础设施建设。实施建设项目“绿色图章”审核制度。加快数字化城管体系建设，提高城市基础设施运行效率。科学规划和开发利用城市地下空间。全力推动以TD网络为依托的无线城市建设，不断提升城市信息化水平。继续抓好城镇“优胜杯”竞赛活动，扶持发展一批具有特色经济实力的重点镇和中心镇。加快新型农村社区建设。对现有“城中村”加快改造，尽快纳入城市发展规划。对产业集聚区、城市新区和中心城市近郊区的村庄，积极推进新型农村社区建设，率先实现城乡一体化。对生态功能区和不宜居住村庄、偏僻山区零散村庄，创造条件建设集中居住区，逐步实施整村搬迁。其他广大农村按照城乡统筹发展要求，不断提升公共服务水平，改善生产生活条件。

    （五）强力推进改革创新，占领科学发展新高地。着眼经济工作全局，努力破解发展瓶颈，深入推进重点领域和关键环节改革创新。继续推进国有经济战略性调整。加快国企改革步伐，基本完成37户国有企业改革任务。进一步加强企业国资监管。全面推进市直机关所属企业脱钩改制。积极推进骨干企业通过增资扩股、股权转让等多种形式重组整合，力争组建一批大型企业集团。鼓励、支持和引导非公有制经济发展，营造各种所有制经济依法平等使用生产要素、公平参与市场竞争、同等受到法律保护的体制环境。创新要素保障机制。认真落实国家鼓励民间投资政策，积极引导社会资本进入基础设施、公用事业、保障性住房等领域。大力发展资本市场，健全多层次资本市场体系。加强金融监管，加大非法融资治理力度。建立健全土地整治、节约集约用地和监督管理机制，保障发展用地需求。大力推进社会事业领域改革。加快经营性文化单位改革，组建市演艺集团，全面推行市、县文化市场综合执法。深化医药卫生体制改革，建立健全覆盖城乡的基本医疗卫生制度。继续深化财税、金融、旅游、城管等领域改革。

    （六）全面提升开放水平，实现科学发展新突破。坚持开放带动，深入开展大招商活动，以开放促改革、促转变、促发展。积极承接产业转移。依托9个省级产业集聚区和7大主导产业，以引进产业链核心环节和龙头企业为重点，突出县（市）区和企业招商主体，瞄准世界500强、国内500强和行业500强，有针对性地承接国内外产业转移。拓宽开放领域。积极推进中建材安阳产业基地和国药集团产业基地建设，力争与央企合作取得实质性进展。加快科技、教育、文化、卫生、体育等领域开放步伐，吸引更多境内外资金进入高技术产业、现代服务业、节能环保产业和现代农业，形成宽领域对外开放格局。提高招商实效。认真组织赴发达国家和地区招商活动。继续抓好已签约重大招商项目跟踪推进，提高项目开工率和投产率。进一步优化投资环境，确保招商项目零障碍进入和实施。

    （七）发展壮大第三产业，打造科学发展新引擎。大力实施“服务业提速行动计划”，着力培育文化旅游品牌，打造国内外有影响力的旅游目的地，带动第三产业快速发展。繁荣发展文化事业。建设覆盖城乡的公共文化服务网络，推进广播电视“村村通”、“农家书屋”等文化惠民工程。加强文化遗产保护与利用，重点做好马氏庄园、曹操高陵、大运河滑县段等申报全国重点文物保护单位工作。加快发展文化产业。推进文化资源整合，加大文化精品创作力度，加快培育大型文化企业集团。积极推进殷墟国家考古大遗址公园和以周易、三国等文化为主题的园区建设，形成有较强影响力的文化产业品牌。以颛顼帝喾陵祭祖节为载体，搭建民俗文化产业发展平台。推出以反映殷商文化、建安文化等为主要题材的电视剧和演艺剧。办好2011中国·安阳殷商文化旅游节、航空运动国际文化旅游节等活动。积极发展旅游业。继续坚持“三阳开泰”旅游发展思路，进一步加强景区管理和基础设施建设。加快推进旅游集散中心等工程建设，着力打造殷墟、羑里城、红旗渠、林滤山、中国文字博物馆等旅游知名品牌。大力发展现代服务业。抓好市豫北汽配销售市场、物流港等项目建设。加快发展通用航空产业。推动金融、物流等现代服务业扩张规模、优化结构、提升水平，尽快成为新的战略支撑产业。

    （八）突出抓好民生改善，谱写科学发展新篇章。继续实施“十项民生工程”，切实解决好事关人民群众切身利益的现实问题。着力扩大就业。实施更加积极的就业政策，多渠道开发就业岗位。加快农民工市场建设步伐，扶持自主创业和自谋职业。重点解决高校毕业生、农村转移劳动力、城镇就业困难人员、退役军人的就业问题。实现下岗失业人员再就业3万人，其中就业困难群体再就业1万人，应届高校毕业生就业率达到80%，新增农村劳动力转移就业5万人。健全社会保障体系。继续扩大养老保险覆盖面，落实基本养老保险关系跨地区转移接续办法。健全企业退休人员基本养老金、优抚补助对象正常调整机制。继续提高企业退休人员基本养老金水平。扎实做好新农保试点工作。完善城镇职工、城镇居民医疗保险制度和新农合制度。切实解决好失地农民生活保障问题，完善被征地农民社会保障制度。健全社会福利和救助体系，鼓励和支持慈善事业发展，基本完成市儿童福利院主体工程建设。大规模实施保障性安居工程。加快棚户区和农村危房改造，大力发展公共租赁住房。新开工建设经济适用住房27万平方米、廉租住房27.6万平方米、公共租赁住房4000套，改造棚户区20万平方米。全面推进各项社会事业。优先发展教育，抓好教育中长期规划，积极推进义务教育均衡发展，实施职业教育攻坚计划。认真落实人才发展规划纲要。办好第八届全国特种养殖种植技术成果博览会。加快卫生事业发展，巩固完善基本药物制度和9项国家基本公共卫生服务项目，继续抓好重大传染性疾病防控和救治工作。全面完成标准化村卫生室建设，加快市人民医院搬迁，基本完成市中医院病房楼主体工程。继续创建国家卫生城市。落实全民健身条例，推进农民健身工程，全力办好市第十届运动会。进一步深化人口和计划生育综合改革，建立统筹解决人口问题新机制，持续稳定低生育水平。加强和创新社会管理。深入推进社会矛盾化解、社会管理创新、公正廉洁执法三项重点工作。健全信访工作责任制，畅通群众利益诉求表达渠道。牢固树立安全发展理念，坚决遏制重特大事故发生。健全应急管理体系，妥善应对群体性事件和突发公共安全事件。强化食品、药品安全监督管理。加强社会治安综合治理，严密防范、依法打击各种违法犯罪活动，保障人民生命财产安全，切实维护社会稳定。

    加强精神文明建设，积极争创全国文明城市。深入开展“扫黄打非”，加强未成年人思想道德建设。继续做好国防教育和民兵预备役建设，深入开展“双拥”共建活动，巩固发展军政军民团结。广泛听取各民主党派的意见，发挥工会、共青团、妇联等人民团体的桥梁纽带作用。积极做好法律服务和援助。依法保护妇女、儿童合法权益，认真做好老龄和关心下一代工作。继续做好统计、审计、档案、史志、气象、地震、发展研究、外事侨务、民族宗教、红十字等工作。

    四、加强政府自身改革与建设

    坚持以领导方式转变促进发展方式转变，强化“三具两基一抓手”和“两转两提”的要求，加快推进政府自身建设和管理创新，努力建设学习型、服务型政府，更好地为人民服务、对人民负责、受人民监督、让人民满意。

    （一）加快职能转变，不断提升政府创新能力。加快行政管理体制改革，推进政府职能向更加注重强化社会管理和公共服务转变，提高公共服务质量和效率。深化行政审批制度改革，减少和规范行政审批，减少政府对微观经济活动的干预。创新行政服务中心管理机制，做到集中审批、联审联办。加强公务员队伍教育管理，增强服务意识，深入开展争做“人民满意的公务员”活动，努力优化发展环境。

    （二）坚持依法行政，不断提升政府决策能力。自觉接受人民代表大会及其常务委员会的法律监督、工作监督，高度重视人民政协的民主监督和参政议政作用，认真办理人大代表议案、建议和政协委员提案，切实增强依法行政的意识和能力。加强政府法制建设，进一步规范行政执法主体，严格行政执法责任制度和过错追究制度，提高政府公信力。落实廉政建设责任制，严格执行廉洁自律各项规定。坚持依法决策、科学决策、民主决策，进一步扩大政务公开范围，及时把与人民群众利益相关的各类事项向社会公开，让权力在阳光下运行。

    （三）注重实干实效，不断提升政府执行能力。牢牢把握重在运作这一履职要求，在工作落实上狠下工夫，说到做到、说好做好。大力整治文风会风，开短会、讲短话、发短文、少发文。严格控制各类检查评比，从简务实地举办会展和节庆活动，腾出更多的精力深入基层、深入群众，调查研究、解决问题。强化大局意识，坚决破除部门本位主义，大力整治行政不作为、工作不落实现象，确保政令畅通。

    （四）着力民心民生，不断提升政府服务能力。继续办好市长热线和效能监督热线，及时了解民意民声，时刻关心人民群众柴米油盐酱醋茶、衣食住行教业保，恪守为民之责、善谋富民之策、多办利民之事。坚持把群众高兴不高兴、拥护不拥护、赞成不赞成、满意不满意作为评判各项工作的标准，谋划改革发展、实施重大项目一定要使人民群众成为现实受益者，不以牺牲人民福祉为代价。大力倡导勤俭节约、艰苦朴素之风，坚决反对挥霍公款和铺张浪费，把有限资金和资源更多地用在发展经济、改善民生上。

    各位代表！

    历史赋予重任，信心凝聚力量。让我们紧密团结在以胡锦涛同志为总书记的党中央周围，在省委、省政府和市委的正确领导下，坚定信心，大胆开拓，为让安阳人民生活得更好，为加快建设豫北区域性中心强市，为以优异成绩迎接中国共产党成立90周年而努力奋斗！
